# HIV Rev-isited

**DOI:** 10.1098/rsob.200320

**Published:** 2020-12-23

**Authors:** Catherine Toni-Sue Truman, Aino Järvelin, Ilan Davis, Alfredo Castello

**Affiliations:** 1 Department of Biochemistry, University of Oxford, South Parks Road, Oxford OX1 3QU, UK; 2 MRC-University of Glasgow Centre for Virus Research, University of Glasgow, 464 Bearsden Road, Glasgow G61 1QH, UK

**Keywords:** HIV, Rev, human, immunodeficiency, virus

## Abstract

The human immunodeficiency virus type 1 (HIV-1) proteome is expressed from alternatively spliced and unspliced genomic RNAs. However, HIV-1 RNAs that are not fully spliced are perceived by the host machinery as defective and are retained in the nucleus. During late infection, HIV-1 bypasses this regulatory mechanism by expression of the Rev protein from a fully spliced mRNA. Once imported into the nucleus, Rev mediates the export of unprocessed HIV-1 RNAs to the cytoplasm, leading to the production of the viral progeny. While regarded as a canonical RNA export factor, Rev has also been linked to HIV-1 RNA translation, stabilization, splicing and packaging. However, Rev's functions beyond RNA export have remained poorly understood. Here, we revisit this paradigmatic protein, reviewing recent data investigating its structure and function. We conclude by asking: what remains unknown about this enigmatic viral protein?

## 1. Introduction

**Figure 1.**
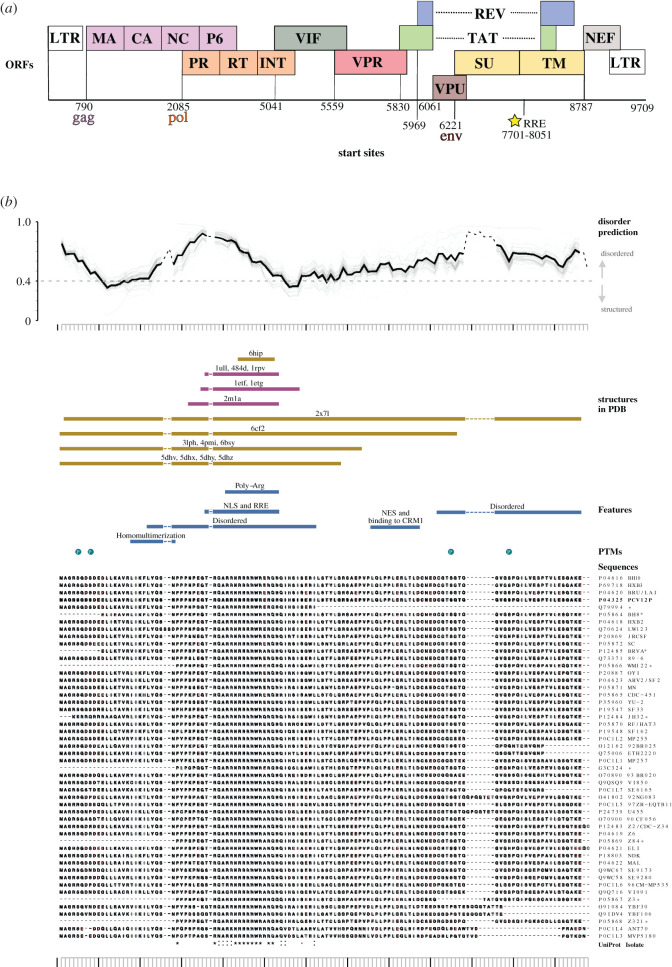
(*a*) Schematic of the HIV-1 genome, which is alternatively spliced. Start sites of open reading frames are numbered according to the NL4-3 HIV-1 sequence. (*b*) Though Rev is partially structured, it is predicted to be highly disordered at the amino acid level (data obtained from IUPred, where a score of greater than 0.4 indicates a high degree of disorder) [2]. Despite its disordered structure, Rev structures have been solved; regions indicated are labelled with Protein Data Base references and coloured for the ARM region (purple) and additional regions (gold). Post-translational modifications (PTMs) and amino acid variants obtained from UniProt are shown (main figure based on variant P04325).

**H**uman **i**mmunodeficiency **v**irus (HIV-1) is a retrovirus that infects CD4+ T-lymphocytes and macrophages, leading to a gradual loss of CD4+ cells and subsequent immunodepression termed acquired immunodeficiency syndrome (AIDS). HIV infects approximately 37 million people globally and is treated using life-long **a**nti-**r**etroviral **t**herapy (ART) [1] that suppresses but does not fully eliminate the virus. Widely used first-line ART include a cocktail of compounds that target the viral enzymes, such as protease, reverse transcriptase and integrase inhibitors [1]. In the search for new therapies, scientists are expanding their interest towards other viral and cellular proteins. HIV-1 expresses 15 proteins from a single approximately 9 kb RNA genome (figure 1*a*) using two different strategies: the synthesis of polyproteins that are processed by the viral protease and alternative splicing. HIV-1 RNA is considered fully spliced when the two large intronic sequences present in the genome are removed. These fully spliced transcripts leave the nucleus using the canonical NXF1-mediated pathway for cellular mRNA export [3]. However, the HIV-1 genome can remain unspliced or undergo a single splicing event, leading to so-called underspliced HIV-1 RNAs. These underspliced transcripts are retained in the nucleus, in an analogous manner to unspliced cellular mRNAs [4,5]. To bypass nuclear retention, retroviral underspliced RNAs harbour regulatory elements that recruit the cellular export machinery either directly or through an adaptor viral protein. For example, Mason-Pfizer monkey virus (MPMV) RNA harbours a **c**onstitutive **t**ransport **e**lement (CTE) that allows direct recruitment of TAP/NXF1 export machinery [6]. By contrast, underspliced HIV-1 RNAs contain an RNA structure, known as the **R**ev **r**esponse **e**lement (RRE), that recruits the viral export factor Rev [7]. Rev is expressed from a fully spliced viral RNA and binds to the RRE to elicit the export of the underspliced viral (v)RNAs to the cytoplasm through the recruitment of host factors. The critical roles of Rev in HIV-1 gene expression extend beyond RNA export and include RNA splicing, stability and translation. How Rev influences these processes, however, remains poorly characterized. Research into Rev has been held back by several technical difficulties, including low expression levels in infected cells and difficulties in expressing tagged Rev from the viral genome. Consequently, researchers have been forced to use systems that do not fully recapitulate physiological HIV-1 infection, often bypassing infection entirely and using cell lines which are not naturally infected by HIV-1. Here, we discuss the functions, structure and protein partners of Rev, examining both the discrepancies reported across varying experimental systems and the data commonly unearthed across them in the light of more recent research. We also discuss which mysteries of this enigmatic protein remain unsolved.

## 2. The knowns of the HIV-1 Rev protein

### 2.1. The architecture of Rev

**Figure 2.**
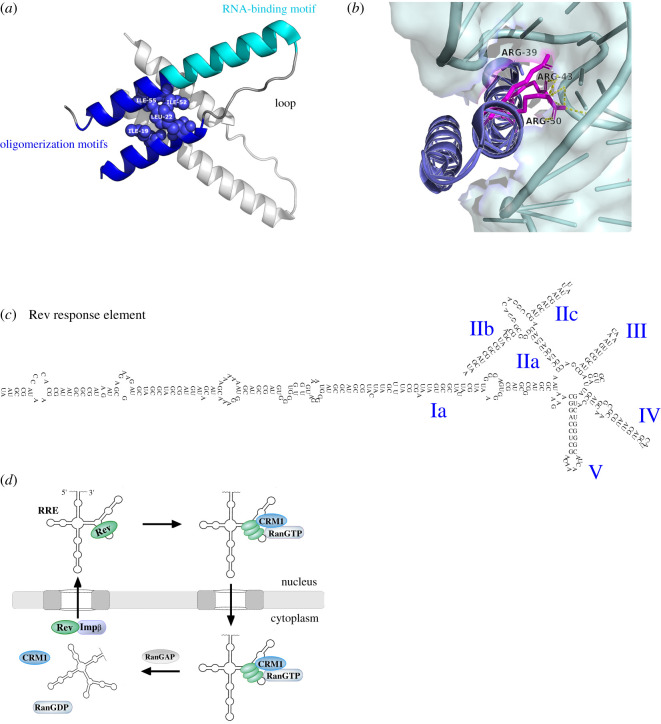
(*a*) The Rev N-terminal domain is composed of a helix–loop–helix motif and dimerizes in a hashtag motif. An arginine-rich RNA-binding motif is flanked by oligomerization motifs on both helices, which allow Rev to multimerize. NTD helices are stabilized by core hydrophobic interactions; some contributing residues are labelled (PDB: 6BSY). (*b*) A Rev dimer was crystallized in complex with the RRE; Rev binds stem IIB of the RRE using its arginine-rich motif, which makes contacts with the negatively charged RNA backbone (PDB: 4PMI [13]). (*c*) The structure of the 351-nucleotide Rev response element; regions are named using Roman numerals. (*d*) The canonical cycle of Rev exporting underspliced viral RNA to the cytoplasm. Rev binds the Rev response element on underspliced RNA at stem IIB, multimerizes and recruits CRM1 and RanGTP to cross the nuclear pore complex. In the cytoplasm, this complex can dissociate by RanGAP promoting RanGTP hydrolysis, freeing viral RNA. Rev can re-enter into the nucleus by binding importin-β.

Rev was discovered through mutations of the overlapping HIV-1 *tat* gene, which specifically increased the production of fully spliced viral RNA and eliminated production of underspliced transcripts [8]. These effects could not be reversed by *in trans* complementation with Tat, suggesting that they were Tat-independent [9]. They were attributed to a novel HIV-1 gene, initially termed *trs/art*, later named **R**egulator of **e**xpression of **v**irion proteins (Rev). Rev comprises 116 amino acids and is highly disordered (figure 1*b*). It harbours a folded N-terminal domain (NTD) and unstructured C-terminal domain (CTD). The 65-residue NTD adopts an anti-parallel helix–turn–helix organization and encodes an arginine-rich RNA-binding motif (ARM) encompassing a nuclear/nucleolar localization signal (NLS) and two flanking oligomerization motifs, which allow Rev to multimerize (figure 1*b*) [10]. Indeed, Rev has a high capacity for self-association and is shown in crystal structures to do so using three types of homotypic interactions termed A–A, B–B and C–C interfaces [11–13]. A–A and B–B interactions occur when same-face α-helices associate in a V motif (figure 2*a*) and exhibit some degree of structural malleability. C–C pairings occur at the loop ends of α-helices by a proline interaction and exhibit restricted flexibility [11–13]. The disordered nature of the Rev CTD has made it challenging to characterize biochemically and structurally, although it has been proposed to fold into β sheets in certain contexts such as filament formation [12,14,15]. Notably, the CTD encodes a leucine-rich nuclear export signal (NES), sometimes referred to as the ‘activation domain’. Rev employs its NES and NLS to traverse the nuclear pore and move between the nucleus and cytoplasm. This shuttling ability is critical to allow Rev to export underspliced viral RNAs and then return to the nucleus [16].

### 2.2. Rev specifically exports underspliced viral RNA from the nucleus

Following reverse transcription, HIV-1 proviral DNA integrates into the host cell genome. Production of HIV-1 RNAs is then mediated by the host RNA polymerase II giving rise to over 100 different RNA species by alternative splicing [17]. The approximately 9 kb unspliced genomic viral RNA encodes Gag and GagPol polyproteins, which are the structural and enzymatic proteins required for virus formation and propagation (figure 1*a*) [18]. The approximately 4 kb singly spliced species encode for the Envelope protein as well as the auxiliary factors Vpr, Vpu and Vif, while the approximately 2 kb fully spliced RNAs encode for regulatory Tat, Rev and Nef proteins (figure 1*a*) [18]. In the early phase of infection, underspliced RNAs are retained in the nucleus and eventually degraded as they are recognized by the host machinery as ‘immature’ [19]. Conversely, *rev* and the other fully spliced mRNAs are exported to the cytoplasm as they are recognized as ‘mature’ transcripts [3,20]. Once Rev protein is produced, it is imported into the nucleus by direct interaction with cellular importins β, transportin, importin 5 and importin 7 through its NLS [21,22]. Ribosome assembly factor B23 has been shown to aid in this import [23,24]. In the nucleus, Rev recognizes specifically a 351-nucleotide secondary structure present in the second intron of underspliced (approx. 9 and approx. 4 kb) viral RNAs, known as RRE [25–33]. Rev interacts with the RRE with high affinity (reported *K*_d_ = 0.3–5 nM), using the arginine residues of the ARM motif aligned in an α-helix which are projected to and inserted into a major groove in the RNA (figure 2*b*) [29,31,34–37]. How Rev assembles on the RRE remains unclear. Some groups postulate that Rev typically binds the RRE as a monomer and oligomerizes thereafter in a cooperative manner along lower affinity sites [28,31,35,36,38–42]. Other groups suggest that Rev exists as a multimer in solution and binds the RRE as such, where gel shift and fractionation experiments show Rev–RRE interacting at distinct stoichiometries [10,21,29,30,34,43]. Technological advances have led to a series of recent Rev and RRE structures, which provide clues to Rev–RRE stoichiometry. 3D small-angle X-ray scattering reconstructions show that the RRE adopts an ‘A-like’ topology [44], where the primary binding site for Rev on stem loop IIB maps to one of the ‘A’ legs, and site IA, a posited secondary Rev-binding site [40,45], maps to the other leg (figure 2*c*). These sites are separated by approximately 55 Å, which matches the span of a Rev dimer [44]. It has been posited that a Rev dimer first binds the RRE at these sites; when either leg of the ‘A’ shape is removed, the RRE is non-functional, supporting this model [46]. Similarly, when the ‘crossbar’ of the A is extended, spanning greater than 55 Å, binding of Rev to the RRE is strongly impaired [46]. A different model envisions two Rev dimers associating through a C–C interface, each dimer binding the RRE separately at sites stem IIB stem IA [12]. This model is supported by a new tetramer crystal structure, which indicates that B–B Rev dimers bind through C–C interfaces to form tetramers, which bind stem IIB and IA sites [47]. Nucleotides G47, G48 and A73 in stem loop IIB of the RRE form non-canonical base pairs that present a wider groove to facilitate Rev binding. Crystal structures have identified that these ‘wobble’ pairings probably help to orient the interaction of a second Rev monomer, allowing it to reach additional junction sites along stem II [13]. Accordingly, mutation of these nucleotides reduces Rev-stem loop IIB interaction, increasing the dissociation constant by over 2 µM in the case of A73 [37]. Regardless, Rev oligomerization appears to be a pre-requisite for successful RNA export since (i) Rev mutants unable to oligomerize are defective for export [39,43,48,49] and (ii) stem loop IIB alone is insufficient to trigger RNA export, suggesting a requirement for oligomerization along low-affinity binding sites [40,50].

After Rev binds the RRE, host cell exportin Chromosome Region Maintenance Gene 1 (CRM1, also known as XPOI) and RanGTP are recruited to the Rev NES [51,52]. Rev appears to interact with CRM1 non-canonically; a recent electron microscopy structure indicates that when CRM1 interacts with Rev, it does so as a dimer, binding the Rev NES [53]. Crystal structures show that Rev NES binds CRM1 in a linear, unorthodox fashion, where the NES is spaced out using proline residues to reach all five hydrophobic binding pockets of CRM1 [54]. It also appears that RRE-bound Rev dimers spatially orient their CTDs to optimally recruit CRM1 [47]. In this RNA-bound state, Rev's NLS is occluded while the NES remains accessible, facilitating CRM1 recruitment and export [21]. It remains poorly understood if additional host cofactors contribute to Rev/CRM1-mediated export. However, recent data posit a string of proteins which may be part of this complex, including phosphoproteins [55], RNA helicases [56,57], nucleoporins [58] and additional factors [59–62], some of which will be discussed in later sections. The Rev-viral RNA–CRM1–RanGTP complex then traverses the hydrophobic channel of the nuclear pore and, once in the cytoplasm, disassembles upon RanGTP hydrolysis. Liberated viral RNAs can then be translated and Rev can be recycled back to the nucleus by importin recruitment (figure 2*d*), which has been shown to occur exclusively following RNA dissociation [21]. In this way, Rev tightly couples the transcription of viral RNAs with their nuclear export, enabling expression of all viral genes in the right quantities and at the right time.

### 2.3. Rev drives underspliced HIV-1 RNAs towards the CRM1 export pathway

Rev reduces the levels of cellular and viral RNAs exported by CRM1-independent pathways by blocking TAP/NXF1, the main cellular pathway that exports the bulk of cellular mRNAs. Taniguchi *et al.* [63] showed that Rev inhibits TAP/NXF1-specific export of RNAs containing the RRE. Overexpression of TAP components decreased the levels of underspliced viral RNA, an effect that could be reversed by Rev overexpression [63]. This suggests that Rev downregulates the TAP/NXF1 pathway to bypass cellular checkpoints that induce the degradation of underspliced viral RNA. Since fully spliced viral mRNAs such as *rev* use TAP/NXF1 to be exported, accumulation of Rev protein may lead to the suppression of TAP/NXF1 export pathway to increase the ratio of underspliced to spliced transcripts in the cytoplasm. Accordingly, Rev was proposed to interfere with the association of TAP/NXF1 components, such as ALY/REF, with RNA [63]. Although the exact mechanism remains unknown, Taniguchi *et al.* [63] propose a model by which RNA-bound Rev may interact with the nuclear **c**ap-**b**inding **c**omplex (CBC) to inhibit its interaction with Aly/REF and subsequently suppress downstream TAP/NXF1 export. Rev can indeed bind subunits of the nuclear CBC [61], which lends credence to this model.

### 2.4. Rev stabilizes viral RNAs

In addition to stimulating nuclear export, Rev may serve several post-transcriptional roles to promote HIV-1 expression. For example, Rev binding to the RRE appears to overcome the inhibitory effects of instability (INS) sequences in HIV-1 RNAs. These INS regions have been identified in the *env, gag* and *pol* coding regions and promote nuclear retention, instability and reduced polysome loading of viral RNA [64–69], independently of splicing. INS regions are AU-rich, which confers a different codon usage to that of human mRNAs [70–72]. The presence of non-optimal codons promotes mRNA instability, likely due to ribosome stalling and inefficient translation at these sites. The mutagenesis of an AU-rich INS site in *gagpol* increases protein expression by ameliorating steady-state mRNA levels [73,74], illustrating the effects of these regions and the importance of codon optimality. High A and low C content produces a codon bias on HIV-1 RNA that may also decrease protein expression because of a lack of cognate tRNAs in steady-state cellular conditions, which induces long ‘waiting times’ for the ribosome to engage with the correct tRNA [75,76]. Indeed, circumventing INS sites with codon-optimization was shown to increase protein expression of HIV-1 Env, independently of effects on RNA export or stability [72]. The Rev–RRE interaction seems necessary to counteract INS-mediated effects [64–68], since mutations nullifying INS regions have been shown to switch HIV-1 protein expression from Rev-dependent to Rev-independent [73,74,77]. How Rev helps to overcome instability signatures is yet to be fully elucidated. However, it has been demonstrated that enhanced expression of codon-optimized *gag* and *pol* genes results predominantly from an increase in cytoplasmic mRNA [74,77]. When cells were pre-treated with leptomycin B, an inhibitor of Rev–RRE export partner CRM1, expression of Rev-dependent HIV-1 proteins was significantly reduced. Expression of codon-optimized counterparts, however, was not affected [76]. This suggests that codon-optimization allows HIV-1 RNAs to use other export pathways, triggering this increase in cytoplasmic RNA. It is therefore likely that INS regions cause nuclear retention of HIV-1 RNAs, which Rev–RRE binding and export is necessary to overcome. It has also been proposed that certain host proteins can bind AU-rich INS sequences and that these interactions regulate HIV-1 RNA stability [78–82]. Rev interacts with some of these cellular factors and it is plausible that by engaging with them, Rev interferes with their regulatory activity on HIV-1 RNA. In summary, Rev emerges as regulator of viral RNA stability by counteracting the inhibitory effects of INS regions.

### 2.5. Rev inhibits cellular splicing

Rev may inhibit splicing in an RRE-dependent manner. The ARM peptide of Rev was found to inhibit splicing of RRE-containing RNA up to 15-fold more than control RNA lacking the RRE [83]. Incubation of the Rev ARM with a β-globin-RRE pre-mRNA at different time points showed that splicing was only inhibited when Rev was added early, suggesting that the Rev/RRE interaction interferes with initial spliceosome assembly on the RNA [83]. Fractionation and sucrose centrifugation of these splicing extracts revealed that the addition of Rev to RRE-containing pre-mRNA removed the 60S peaks corresponding to fully assembled spliceosomes [83], and caused an accumulation of a 45-50S splicing-deficient complex. The same group discovered that the Rev ARM blocked binding of U4/U5/U6 tri-snRNP in an RRE-dependent manner [84]. While it is not known how Rev exerts these effects, it may interact with host cell splicing factors to do so. A recent study used a genome-wide CRISPR/Cas knock-out approach to identify host cell proteins responsible for HIV-1 RNA nuclear retention. The majority of the resulting hits were host cell proteins involved in pre-mRNA splicing and associated with the spliceosome [85]. Perhaps Rev suppresses formation of the early spliceosome by interacting with these factors, ultimately promoting intron retention. One example of this is SF2/ASF, an essential splicing factor that binds to RNAs with a 5′ splice site to aid U1 snRNP docking [86]. SF2/ASF binds the Rev-bound RRE *in vitro* and its overexpression can inhibit Rev function and HIV-1 gene expression in a dose-dependent manner [86]. It also regulates HIV-1 gene expression [87–89]. It is plausible that Rev sequesters this splicing factor, and possibly other spliceosome-associated components, to prevent spliceosome recruitment. Importantly, SF2/ASF overexpression has been shown to alter the alternative splicing pattern of HIV-1 [90]. Hence, Rev may bind this factor in infected cells to ensure optimal stoichiometry of HIV-1 RNA forms. At a global level, HIV-1 infection increases the proportion of introns within cellular RNAs in primary T cells [91], aligning well with this inhibitory activity of Rev on SF2/ASF. Collectively, these posited roles paint a picture of Rev working against host cell regulation to repress splicing and promote the expression of underspliced HIV-1 RNA.

### 2.6. Rev promotes translation of viral RNA

It has been proposed that Rev influences the translation efficiency of viral RNA. Early research highlighted disproportionate increases in viral Envelope protein expression relative to total mRNA levels in Rev and Tat-transfected cell lines [92]. Additional studies confirmed large discrepancies between cytoplasmic *gag* mRNA and protein levels in the absence of Rev [93–96], providing the first clue that Rev may affect translation. Moreover, Rev appears to facilitate association of viral RNA with polysomes. One study found that 90% of cytoplasmic, singly spliced *env* RNA associated with monosomes when infected with HIV-1 lacking Rev (HIV-1_Rev(-)_), whereas over 75% associated with polysomes when infected with wild-type Rev HIV-1 [94]. Conversely, polysomal association with fully spliced viral RNAs such as *tat* was unaffected by the presence or absence or Rev, indicating efficient assembly of ribosomes onto these viral RNAs in a Rev-independent manner. Interestingly, Rev-dependent association of underspliced viral RNA with polysomes was found to be dependent on the presence of the RRE, suggesting the need of Rev/RRE interaction for this phenomenon to occur [93]. However, tethering of the leucine-rich domain of Rev, which interacts with CRM1, to HIV-1 RNA allowed efficient Gag production in the absence of Rev, indicating that RNA export is the predominant function of Rev [97]. These results also suggested that CRM1-mediated export, instead of Rev itself, may be sufficient to enable HIV-1 RNA downstream translation through an export/translation coupling mechanism. Intriguingly, a conserved Rev-binding site was discovered in the 5′ UTR of HIV-1 RNA, overlapping with the loop A of the packaging signal (SL1) [98]. Rev enhanced the translation of loop A containing reporters *in vitro* translation [99]. However, these findings were not recapitulated by the same group in COS-1 cells [100]. Whether Rev controls HIV-1 RNA translation remains ultimately unclear.

### 2.7. Does Rev regulate packaging of HIV-1 genome into virions?

The presence of a packaging signal in the 5′ untranslated region (UTR) of HIV-1 genomic RNA does not suffice for efficient assembly of the viral RNA into viral particles. The interaction of Rev with the RRE is also proposed to contribute to viral RNA packaging [101–104]. The ratio of genomic RNA in virions over the cytoplasm was measured using an HIV-1 chimeric construct containing the RRE in the presence or absence of Rev [105]. Lack of Rev induced a decrease in genome packaging of 10-fold when compared with conditions where Rev was present [105]. However, the RRE–RNA construct used in this study lacked the original INS present in the HIV-1 genome, and thus probably does not display the same dependency on Rev for nuclear RNA export as wild-type HIV-1 genomic RNA. The same group, therefore, repeated this work, using an almost full-length HIV-1_Rev(-)_ expression plasmid [106]. They reported that Rev induces a 4500-fold increase in HIV-1 genome packaged into virions, while it only increases cytoplasmic levels by 5-fold [106]. However, the same enhancement in RNA assembly was observed when a chimeric HIV-1_Rev(-)_ genomic RNA including MS2 stem-loops was co-transfected with MS2-TAP [106]. This suggests that is not necessarily Rev, but the export process itself which boosts the packaging of RNA genomes. Regardless, Rev–RRE interaction appears to be more efficient at promoting packaging than TAP, leaving room for potential direct roles of Rev in packaging [104,105]. The mechanistic details of how Rev confers increased genome encapsidation remain unknown. It is possible that certain export pathways lead to the formation (or avoidance) or specific ribonucleic–protein complexes which may promote or hinder downstream packaging. Indeed, helicase DDX24 has been shown to directly interact with Rev and increase RNA packaging only in the context of Rev/RRE export [107].

The Rev-binding site situated in the HIV-1 5′ UTR (loop A of SL1) is also proposed to stimulate viral RNA assembly into viral particles [98,100,108]. However, recent reports suggest that HIV-1 RNA is more efficiently packaged as a dimer than as a monomer [104,109]. Dimer formation required base pairing across the 5′ UTR, including the Gag AUG start codon and the dimerization initiation site (DIS) [104]. It is thus possible that the loop A, situated between DIS and the Gag AUG initiation codon, contribute to dimer formation. Therefore, whether the loop A contributes to viral RNA assembly into viral particles through a Rev-dependent or independent mechanism remains controversial.

### 2.8. The Rev–host interactome

**Figure 3.**
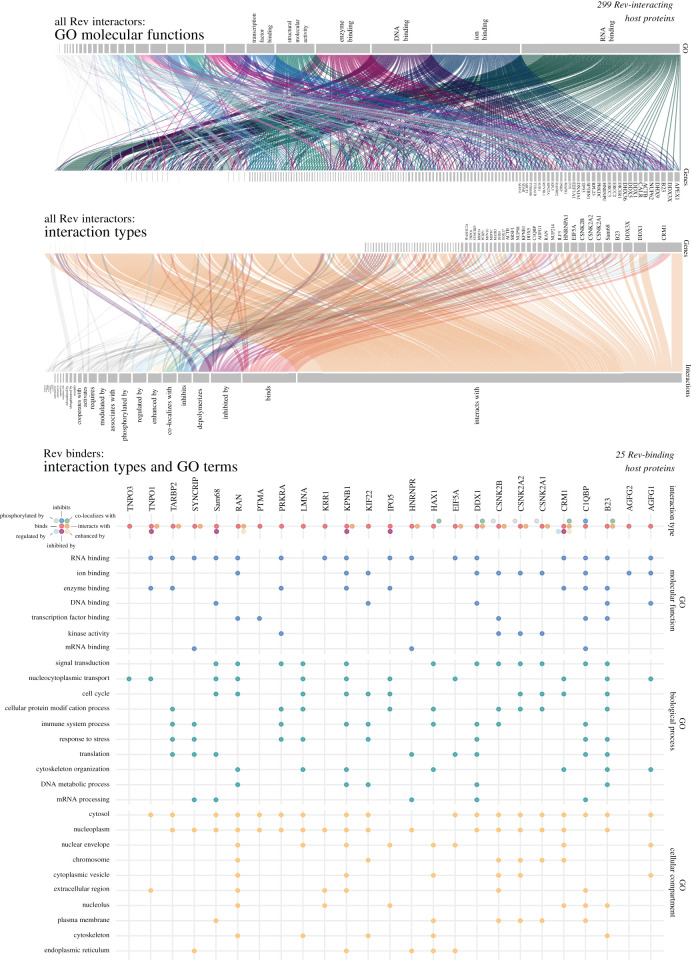
Almost 300 Rev–host protein interactions have been reported in the NCBI HIV-1 interaction database. Of these interactions, many cofactors appear to be nucleic acid binders, as shown by GO slim terms. In the second panel, the type of interaction reported is listed alongside the most reported proteins. Most of these cofactors are simply reported to interact with Rev, though some are known to be inhibitors or enhancers, etc. The third panel explores the interactions of 25 reported Rev cofactors, listing their known GO molecular functions, biological processes and cellular compartments in more detail.

**Figure 4.**
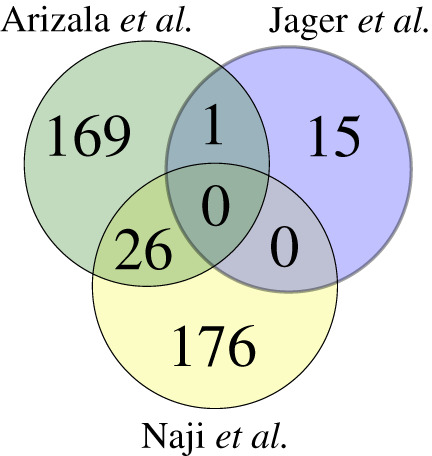
The overlap of host proteins identified to interact with Rev from three available datasets discovered using IP and MS. For Naji *et al.* [111], the top 250 candidates were considered as cited in the paper; for Jager *et al.* [114], proteins from both cell types were considered. For Arizala *et al.*, reported proteins were normalized to HGNC IDs using the R package biomaRt [120] and pseudogenes/proteins which could not be mapped were filtered out.

HIV-1 cannot encode all the machinery required for its replication and spread. Thus, it is heavily reliant on host cell resources. One prevalent strategy used by viruses to hijack cellular resources is to express viral proteins that interact with and recruit key cellular factors. By knowing which cellular factors Rev interacts with, it is thus possible to obtain deeper mechanistic insights into its regulatory roles. A common approach for discovering protein–protein interactions (PPIs) globally involves immunoprecipitation (IP) followed by mass spectroscopy analysis (MS). This approach has been employed to reveal the interactomes of HIV-1 proteins [110–114]. Few studies have focused on Rev interactions using this approach [111,114–116], and have enriched our current knowledge of the Rev–host protein interactome considerably, which to date comprises almost 300 interactors (figure 3) [111,117]. However, a number of technical limitations forced researchers to use non-physiological systems to study Rev, which implies that many of the interactions identified may not take place when Rev is expressed at physiological levels and concomitantly interacting with the viral RNA in infected cells (table 1). Furthermore, the cell system, cofactors, controls, tags and proteomic quantification used in these studies vary widely. This is important because even small changes in the experimental conditions can have a profound impact in the observed interactions. For example, addition of Mg^2+^ was recently reported to impact the structural dynamics of the RRE and potentially Rev binding [118]. It has also been found that the importin which mediates Rev nuclear import is cell-type specific and will therefore differ between cell systems [119], which may impact downstream Rev function. It is difficult, as a result, to collate published datasets obtained by employing different systems. Another important problem regarding interactome analysis is the quantitation, controls and statistical analysis of proteomics data. The lack of a universal, standardized data analysis pipeline and the differences in experimental set-up makes it extremely difficult to compare different datasets. The limitation of self-defined scoring is that results depend somewhat on arbitrary assignments for significance. For example, one study identified only 19 overlapping protein–protein interactions between their results and VirusMint, a database of virus–host cell PPIs, but this increased to 67 when the MS processing threshold score was altered [114]. Such subjective quantitation parameters are problematic when cross-referencing large datasets produced by different groups. As a result of these obstacles and differing IP systems, overlap between reported Rev interactors is limited (figure 4) [59,114,116,121,122]. The lack of overlapping can also be explained by the expression of Rev in non-physiological levels and outside the natural infection context. To circumvent these limitations, future work should focus on the biochemical characterization of Rev expressed from an HIV-1 provirus (fully infective HIV-1 construct or replicons), ideally tagged to facilitate biochemical characterization under stringent conditions. The use of chimeric HIV-1 viruses or replicons allows experiments to be performed in more physiological models such as T CD4 lymphocytic lines and primary cells. Such an approach would ensure that Rev and its HIV-1 RNA substrates are produced at physiological levels and in the correct stoichiometry.

**Table 1.** Technical challenges.

experimental consideration	technical challenge	experimental compromise	experimental issue
Rev is an RNA-binding protein	Rev can establish strong interaction with RNA	RNase treatment is critical to differentiate direct from indirect interactions in protein–protein interaction analysis	Rev can be co-purified with proteins that do not interact directly with it through RNA bridges; hence, protein–protein interaction studies lacking RNases should be considered against this background
Rev gene is entirely overlapped by other viral genes (figure 1*a*)	Rev is, therefore, difficult to genetically manipulate, to clone or tag, in the context of a replicon or virus	full length or selected regions of Rev are often ectopically expressed and, in several cases, it is not co-expressed with HIV-1 RNA	ectopic expression may result in artificial localization and non-physiological expression levels
			expression of select motifs may lead to non-native folding conformations and behaviourRev function relies on its multimerization of HIV-1 RNA; in the absence of HIV-1 RNA, Rev are may thus assemble in non-physiological complexes; moreover, Rev and underspliced HIV-1 RNA are at a given stoichiometry that is difficult to recapitulate using plasmids
Rev is a low abundant HIV-1 protein	Rev is difficult to pull down and isolate at sufficient concentration for biochemical characterization	Rev is typically overexpressed	Rev expression at non-physiological levels is likely to lead to non-native behaviour
			without co-expression of the viral RNA under physiological stoichiometry, Rev may establish artificial interactionsantibodies available against Rev do not allow efficient and selective immunoprecipitation; hence, tags might be required
HIV-1 infects and propagates in CD4+ lymphocytic cells	T-lymphocytes are difficult to transfect	Rev is typically expressed in uninfected non-relevant cell lines (e.g. HEK293T, HeLa), and cell extracts	the proteome of a CD4- cell is expected to differ from that of a CD4+ lymphocytic counterpart; hence, Rev may establish non-physiological interactions in HIV-1 unrelated cell lines
			HIV-1 infection also causes a profound remodelling of the cell proteome and transcriptome; hence, Rev complexes detected in uninfected conditions might be non-functional or just not exist in infected cells

Despite the challenges of uniting various interactome data, there is a set of proteins identified consistently in a number of different studies, imbuing them with credibility [117,123,124]. The known Rev interaction network is explored in figure 3, showing which types of interaction have most often been identified and the GO molecular functions of the interactors. The evidence behind the interaction of some of these widely reported Rev factors is discussed below.

DEAD (Asp–Glu–Ala–Asp)/H box RNA helicases have been functionally implicated in all steps of RNA metabolism as well as ATP-dependent RNA duplex unwinding. Several DEAD/H box proteins interact with Rev, most prominently, DDX1. DDX1 was initially identified to bind a motif of Rev in yeast-two hybrid screens and then confirmed to interact with Rev using protein overexpression and co-immunoprecipitation [62,125]. DDX1 silencing reduced the expression of Gag from a Gag–RRE expression vector by greater than 85% in the presence of Rev, signifying that DDX1 may promote Rev–RNA export [62]. The observation of Rev–RRE binding using total internal reflection microscopy and mobility shift assays confirmed this, revealing that DDX1 enhances higher-order Rev/RRE stoichiometries [41,42]. Although no strict functional mechanism is yet determined, a recent model acknowledging these data proposed that DDX1 may act as an RNA chaperone, remodelling stem IIB of the RRE to facilitate Rev binding [42]. DDX3 is another DEAD/H box helicase linked to HIV-1: it is a nucleocytoplasmic shuttling protein able to co-precipitate both Rev and CRM1, leading to the suggestion that it participates in Rev/RNA/RanGTP/CRM1 complex export [126]. Indeed, knock-out of endogenous DDX3 can suppress RRE–RNA export 13-fold in a Rev-dependent manner, as measured by Gag expression [126]. Importantly, this effect is not observed when the RRE is replaced with a different transport element. Additionally, Northern blotting analysis showed that DDX3 significantly increased levels of *gagpol* mRNA in the cytoplasm transcribed from a Gagpol-RRE expression vector, confirming that it influences Rev-dependent RNA export [126]. Other helicases, including DDX5, DDX17 and DDX21, interact with Rev and enhance Rev-mediated RNA export [127,128]. It is thus plausible that these helicases cross-talk to influence viral RNA export cooperatively. The exact functional mechanisms underpinning Rev regulation by helicases, however, await discovery.

CRM1 was one of the earliest Rev interactors identified. It was initially shown to bind Rev through separation of nuclear envelope proteins by gel electrophoresis, followed by treatment with radiolabelled Rev protein [129], which revealed a band of approximately 110 kDa. Similarly, the radioactively labelled Rev NES was incubated in cell extracts and interacting proteins were eluted and separated by gel electrophoresis, which also highlighted an approximately 110 kDa band, suggesting that this unknown protein could bind Rev at its NES. This ‘p110’ mysterious protein was later identified as an exportin, named CRM1 [130], which is inhibited by leptomycin B [131]. This novel association linked Rev to a nuclear translocation system for the first time and began to reveal the mechanics of Rev transactivation. Elegant experiments by Fukuda *et al.* [130] expressing the NES peptide of Rev in the nuclei of fibroblasts revealed that it was rapidly exported to the cytoplasm. Cells pre-treated with leptomycin B, however, inhibited this nuclear export, corroborating the notion that CRM1 was probably responsible for Rev translocation across the nuclear pore. Mutations of the homologue Crm1p in yeast were similarly shown to reduce Rev transactivation activity. Moreover, the ability of Crm1p to interact with both nuclear pore proteins and Rev was reported in this system, providing further evidence that CRM1 guides Rev across the nuclear envelope [132]. Interestingly, Ristea *et al.* [133] found that Rev and CRM1 colocalize in the nucleolus. This colocalization is dependent on a functional Rev NES, suggesting that both proteins are directly interacting. In the same study, overexpression of CRM1 appeared to reduce Rev-mediated RRE(+)–RNA export in a dose-dependent manner, which the authors suggest may occur due to CRM1 sequestering Rev in the nucleolus. It is notable, however, that overexpressed CRM1 is dislocated, and this aberrant distribution might be the cause Rev of dysfunction.

Eukaryotic Initiation Factor 5A (eIF5A) is an essential protein that facilitates translation elongation of polyproline regions and prevents ribosomal stalling [134]. It can bind specifically to the NES of Rev [135,136]. Non-functional eIF5A mutants that retain the ability to bind Rev hamper the export of Rev–CRM1 complexes to the cytoplasm [135,137]. Indeed, T CD4 lymphocytic cell lines overexpressing these mutants failed to sustain HIV-1 replication efficiently [136]. A pool of eIF5A localizes at the periphery of the nuclear pore complex [137] and has been found to interact with nucleoporins [60]. Moreover, recombinant GST-Rev protein export to the cytoplasm was abrogated by using antibodies against eIF5A [138]. Taken together, these data suggest that eIF5A plays a critical role in the Rev/CRM1-mediated export of HIV-1 underspliced RNAs. However, several groups failed at confirming the existence of an interaction between EIF5A and Rev [21,139], and thus whether this complex plays a physiological role in HIV-1 infection remains controversial.

### 2.9. Rev and its interactions with cofactors are HIV-1 drug targets

Rev is critical for HIV-1 gene expression and, therefore, represents a potential anti-viral target. Despite this, there are currently no Rev-based therapeutics in clinical use. It has long been known that dominant negative mutants of Rev can abrogate wild-type Rev function in lymphocytic cells [140,141]. For example, ‘Rev M10’ contains two point mutations in the Rev NES which completely abrogates its transactivation function, while competing with wild-type Rev for binding to the RRE [48]. M10 has been investigated in clinical trials [142–144]. However, it is challenging to deliver into cells, and resistant strains of HIV-1 with altered RREs arose after constitutive M10 expression [145].

Other therapeutic approaches target the Rev/RRE interaction itself using small molecules. An early iteration of this approach used aminoglycoside antibiotics which specifically bound to the RRE at the Rev-binding site [146], blocking Rev's interaction and inhibiting HIV-1 gene expression [147]. Since then, a series of small molecules able to inhibit Rev/RRE association have been reported, including aminoglycosides, antisense nucleic acids [148,149], synthetic diphenylfuran cations [150,151], RNA aptamers [152,153], metallopeptides [154,155] and several pre-existing drug compounds [156]. Several of these agents bind to the RRE synonymously to Rev, inserting basic regions into the same wobble-base groove in the RRE. Peptide ligands have been developed which similarly adopt the same α-helicity as the Rev ARM; in some cases, these ligands are able to bind to the RRE with higher affinity than Rev itself (about sevenfold) and can successfully block HIV-1 replication [157–161]. Other small molecules, including 8-azaguanine, suppress viral gene expression by redirecting localization of Rev to the cytoplasm, impairing its function [162]. While these agents are anti-viral, off-target effects often render them toxic for human cells. Moreover, those that rely on structure specificity inadvertently apply a selection pressure for RRE and Rev, leading to mutations that provide resistance [163]. It also remains challenging to deliver these treatments to target cells.

As CRM1 is required to escort RNA-bound Rev across the nuclear envelope, blocking the CRM1/Rev interaction is a potential anti-viral opportunity. It is well established that CRM1-inhibiting drugs, such an anti-fungal agent leptomycin B, can restrict HIV-1 replication [131,164]. However, CRM1 typically exports host proteins and is instrumental for exporting ribosomal subunits (for a comprehensive review, the reader is pointed to Okamura *et al.* [165]). Therefore, CRM1 inhibition affects downstream targets and the cellular environment and, indeed, leptomycin B is toxic to human cells. However, small molecular inhibitor KPT-185 was able to restrict both HIV-1 replication and AIDS-induced primary effusion lymphoma in primary cells by blocking Rev/CRM1 interactions [166] and elicited cytotoxic effects only at concentrations 850-fold higher than the active concentration [166].

Targeting other Rev–host cofactor interactions is similarly difficult but not impossible: Campos *et al.* developed ABX464 [167], a drug that restricts HIV-1 replication in mice. ABX464 binds to and stabilizes the CBC complex, enhancing RNA export by the TREX export pathway of fully spliced RNAs, antagonizing Rev posited inhibition of this pathway [63]. Importantly, while it changes the levels of spliced/unspliced viral RNA, ABX464 does not affect cellular RNA [167]. It has since completed three phase II clinical trials and has successfully restricted HIV-1 replication *in vivo.* This highlights the importance of reproducibly defining the Rev interactome: it may unlock dozens of potential therapeutic targets.

## 3. The known unknowns of the HIV-1 Rev protein

### 3.1. The native Rev interactome remains unknown

The role of Rev in RNA trafficking was identified shortly after its discovery in 1986; in the subsequent 30 or more years of research, Rev has continued to pose more questions than answers. For example, we have discussed here the known cofactors of this elusive protein, though the reader may note that much of this research is ‘correlative’ with the underlying molecular mechanism remaining unknown. As Rev is a difficult protein to study (table 1), the number of Rev–host protein interactome studies is limited, and those available have been defined under a broad range of conditions and criteria. It is, therefore, difficult to list interactors validated under common physiological environments. As aforementioned, the overlap between these datasets is low (figure 4). Some researchers have used an approach to examine which host proteins are involved in Rev's RNA export function by capturing the singly or unspliced viral RNA and using MS to examine which proteins are bound [59,121,122]. These studies may also reveal Rev-binding partners. Consideration of these protein datasets corroborate some of the unshared host proteins reported in Rev protein–protein studies, indicating that candidate proteins identified can be true interactors despite the limited overlap between datasets. For example, approximately 30% of the proteins found by Marchand *et al.* [114] are also reported by Naji *et al.* [111]. However, the overlap between these datasets remains modest, and the problem of varying experimental conditions persists.

### 3.2. There is no known function behind Rev nucleolar localization

While Rev participates in nucleocytoplasmic shuttling, it also displays a well-documented tendency to localize in the nucleolus [22,48,120,125,168,169], which is currently unexplained. This has recently been suggested to arise from masking of the NES, as mutation of the NES constrains Rev to the nucleolus. Moreover, Behrens *et al.* [170] have shown that deliberate masking and unmasking of the NLS is able to alter this phenotype. Though the function behind this nucleolar residence is undetermined, Rev mutants that do not localize in the nucleolus are impaired in their ability to export viral RNA [120,168], suggesting it is critical to the Rev functional cycle. Reinforcing this idea, FRET measurements also suggest that Rev dimerization occurs in the nucleolus [171]. Additionally, when Rev and CRM1 are overexpressed separately, they yielded nucleolar and nuclear envelope localization, respectively [166]. However, when both were overexpressed together, CRM1 mobilized to the nucleolus in a Rev-dependent manner. These results suggest that the nucleolus may be an interaction point for both proteins [166]. The roles of Rev nucleolar localization remain under intensive investigation.

### 3.3. The Rev C-terminal domain is structurally unresolved

Some of the challenges of working with Rev have been solved thanks to recent technological advances. For example, Rev tends to aggregate and precipitate [172], and these properties have represented a challenge to resolve its structure [12,172]. In fact, it took 14 years after the initial Rev–RNA NMR models [27] until the first crystal structures of Rev were resolved [11,14]. Thanks to advances in crystallography technologies, the field has since reported invaluable crystal structures of Rev [12,47] and even RRE-bound Rev dimers [13] which have massively contributed to our understanding of the Rev–RRE interaction. However, the Rev CTD still remains mysterious due to its structural plasticity that forces researchers to either delete it or leave this region unresolved in order to obtain structural information [12]. Recent mutagenesis research suggests that the CTD may help stabilize Rev and prevent aggregation [173]. Conversely, an increase in HIV-1 fitness is observed when stop codons are included in the CTD, suggesting it may play an inhibitory role [174]. More work must be carried out to resolve the molecular function of the CTD. As the structural biology field continues to advance, more structures will emerge, and these will probably provide an unprecedented view on the interactions orchestrated by Rev.

## 4. Conclusion and future perspectives

Much is known about the HIV-1 Rev protein, particularly of its essential role in RNA export. However, many questions remain unsolved. It is thus vital that systems and methodologies are established which strive to more closely emulate natural HIV-1 infection and native Rev activity when elucidating the *unknowns.* These improved approaches will hopefully advance us towards understanding the structure, functions and interactions of the HIV-1 Rev protein. In turn, a better understanding of Rev biology will probably open novel therapeutic avenues on the quest to combat HIV-1 infection.
